# Mice Lacking Inositol 1,4,5-Trisphosphate Receptors Exhibit Dry Eye

**DOI:** 10.1371/journal.pone.0099205

**Published:** 2014-06-05

**Authors:** Takaaki Inaba, Chihiro Hisatsune, Yasumasa Sasaki, Yoko Ogawa, Etsuko Ebisui, Naoko Ogawa, Minoru Matsui, Tsutomu Takeuchi, Katsuhiko Mikoshiba, Kazuo Tsubota

**Affiliations:** 1 Department of Ophthalmology, Keio University School of Medicine, Shinjuku, Tokyo, Japan; 2 Department of Rheumatology, Keio University School of Medicine, Shinjuku, Tokyo, Japan; 3 Laboratory for Developmental Neurobiology, RIKEN Brain Science Institute, Wako, Saitama, Japan; 4 Department of Pharmacy, Chiba Institute of Science, Choshi, Chiba, Japan; 5 Calcium Oscillation Project, International Cooperative Research Project-Solution Oriented Research for Science and Technology, Japan Science and Technology Agency, Kawaguchi, Saitama, Japan; Universität Regensburg, Germany

## Abstract

Tear secretion is important as it supplies water to the ocular surface and keeps eyes moist. Both the parasympathetic and sympathetic pathways contribute to tear secretion. Although intracellular Ca^2+^ elevation in the acinar cells of lacrimal glands is a crucial event for tear secretion in both the pathways, the Ca^2+^ channel, which is responsible for the Ca^2+^ elevation in the sympathetic pathway, has not been sufficiently analyzed. In this study, we examined tear secretion in mice lacking the inositol 1,4,5-trisphosphate receptor (IP_3_R) types 2 and 3 (*Itpr2^−/−^;Itpr3^−/−^*double-knockout mice). We found that tear secretion in both the parasympathetic and sympathetic pathways was abolished in *Itpr2^−/−^;Itpr3^−/−^* mice. Intracellular Ca^2+^ elevation in lacrimal acinar cells after acetylcholine and epinephrine stimulation was abolished in *Itpr2^−/−^;Itpr3^−/−^* mice. Consequently, *Itpr2^−/−^;Itpr3^−/−^* mice exhibited keratoconjunctival alteration and corneal epithelial barrier disruption. Inflammatory cell infiltration into the lacrimal glands and elevation of serum autoantibodies, a representative marker for Sjögren’s syndrome (SS) in humans, were also detected in older *Itpr2^−/−^;Itpr3^−/−^* mice. These results suggested that IP_3_Rs are essential for tear secretion in both parasympathetic and sympathetic pathways and that *Itpr2^−/−^;Itpr3^−/−^* mice could be a new dry eye mouse model with symptoms that mimic those of SS.

## Introduction

Because tears keep the cornea and conjunctiva continuously moist, and a reduction in tear volume results in dry eyes (e.g. keratoconjunctivitis sicca), investigation of the regulatory mechanisms underlying tear secretion is crucial for understanding the pathology of ocular systems and for the development of new treatments for dry eyes.

Tear secretion from the lacrimal glands is regulated by two types of nerves: parasympathetic and sympathetic. The activation of parasympathetic and sympathetic nerves predominantly releases the neurotransmitters acetylcholine (Ach) and norepinephrine, respectively [1], [2]. Upon binding to muscarinic acetylcholine receptors, Ach activates phospholipase C and produces inositol 1,4,5-trisphosphate (IP_3_), which in turn triggers intracellular Ca^2+^ release through the IP_3_ receptor (IP_3_R) from the endoplasmic reticulum (ER) in lacrimal gland acinar cells [1]. Stimulation of the α- and β-adrenergic receptors by norepinephrine also induces Ca^2+^ release from internal stores [1], [2]. However, in contrast to the established role of IP_3_Rs in the cholinergic pathway, the Ca^2+^ channels that contribute to Ca^2+^ elevation in the sympathetic pathway are still obscure. It was reported that the activation of α1-adrenergic receptor, a predominant type of adrenergic receptor in lacrimal glands, increases intracellular Ca^2+^ without IP_3_ production, and cyclic ADP-ribose is thought to be involved in the Ca^2+^ increase via the ryanodine receptor–another Ca^2+^ channel on the ER [2]–[5].

To examine the physiological role of IP_3_Rs in the sympathetic pathway of lacrimal glands, we measured tear secretion in IP_3_R-deficient mice (*Itpr2^−/−^;Itpr3^−/−^*), in which several exocrine secretion pathways were disrupted [6], [7]. We found that *Itpr2^−/−^;Itpr3^−/−^* mice show impaired tear secretion via both the parasympathetic and sympathetic pathways and therefore exhibit dry eye. In addition, we detected abnormalities in *Itpr2^−/−^;Itpr3^−/−^* lacrimal gland tissues, such as inflammation, infiltration, and elevated autoantibodies, and these abnormalities mimic human Sjögren’s syndrome (SS). Thus, the *Itpr2^−/−^;Itpr3^−/−^* mouse is a new dry eye animal model caused by disturbed Ca^2+^ signals in lacrimal glands.

## Materials and Methods

### Ethics Statement

All animal procedures in this study were approved by the Animal Experimental Committees at the Institutes of Physical and Chemical Research (RIKEN) -Research Center for Brain Science Institute (BSI) (Permit Number: H25-2-202). All efforts were made to minimize animal suffering. Mice [6] were housed on a 12 h light–dark cycle, with the dark cycle occurring from 8∶00 P.M. to 8∶00 A.M in a specific pathogen-free environment of the Laboratory Animal Facility of the RIKEN Brain Science Institute. In all experimental groups, mice were used at 6–40 weeks of age and 50% were female. Tear collection from mouse eyes was performed under anesthesia with intraperitoneal injection of ketamine and xylazine.

### Immunoblotting

Tissues from the lacrimal glands were homogenized in a solution containing 0.32 M sucrose, 5 mM Tris-HCl (pH 7.4), 1 mM ethylene diamine tetraacetic acid, 0.1 M phenyl methyl sulfonyl fluoride, 10 mM leupeptin, 10 mM pepstatin A, and 1 mM 2-mercaptoethanol (homogenizing buffer). The homogenate containing the lacrimal glands was centrifuged at 1000×*g* for 5 min at 4°C, and the precipitated lacrimal glands were lysed with sample buffer (125 mM Tris-HCl, pH 6.8; 20% glycerol; 4.0% sodium dodecyl sulfate [SDS]; 10% 2-mercaptoethanol; 0.1% bromophenol blue). A total of 50 µg protein was separated by 5% SDS-polyacrylamide gel electrophoresis (PAGE) and transferred to a polyvinylidene difluoride membrane. The membrane was blocked with 5.0% skim milk in 0.05% Tween/phosphate-buffered saline (PBST) for 1 h and probed with the indicated primary antibodies. The primary antibodies KM1112, KM1083, and KM1082 were used to detect IP_3_R1, IP_3_R2, and IP_3_R3, respectively [8]. The Pan-IP_3_R antibody is an antibody that recognizes the consensus epitope of all types of IP_3_Rs [9]. Anti-β-actin antibody (AC-15) was purchased from Sigma (Tokyo, Japan). Incubation of the membrane with the primary antibody was performed for 2 h at room temperature. After washed with PBST, the membrane was further incubated with horseradish peroxidase-labeled secondary antibodies (1∶4000; GE Healthcare, Amersham, UK) for 1 h at room temperature, and the immobilized specific antigen was visualized with the ECL plus detection kit (GE Healthcare).

### Measurement of Tear Secretion

The mice were anesthetized by intraperitoneal injection of 36 mg/kg ketamine (Daiichi Sankyo, Tokyo, Japan) and 16 mg/kg xylazine (Bayer Healthcare, Leverkusen, Germany). Tear production was stimulated by intraperitoneal injection of 3 mg/kg pilocarpine (Santen, Osaka, Japan) or 1 mg/kg epinephrine at 1 min after the anesthesia. Tears were collected for 15 min and the volume was calculated every 5 min during the 15-min duration using 0.5-µL capillary microglass tubes (Drummond, PA, USA). After the measurement, the mice were sacrificed, and the lacrimal glands were extirpated. Then, the lacrimal gland weights were measured, and the mean values were calculated to obtain the average lacrimal gland weight of the mice. The tear secretion volume was adjusted for the weight of the each lacrimal gland.

### Histopathology and Electron Microscopy

For histopathology, the extracted lacrimal glands and conjunctiva were embedded in an optimal cutting temperature compound (Sakura Finetechnical, Tokyo, Japan). Frozen sections (5-µm thick) of the lacrimal glands or the conjunctiva were fixed with 10% formalin neutral buffer solution (Wako, Osaka, Japan) and stained with hematoxylin and eosin or with the periodic acid-Schiff reagent. For electron microscopic observation, a portion of the lacrimal glands was fixed with 2.5% glutaraldehyde in 0.1 M phosphate buffer overnight and was post-fixed with 1.0% osmic acid in 0.1 M cacodylate buffer. The specimens were dehydrated with ethanol and embedded in epoxy resin. The ultra-thin sections (80 nm) were double-stained with uranyl acetate and lead citrate, and were examined under a transmission electron microscope (1200 EXII; JEOL, Tokyo, Japan).

### Immunohistochemical Analysis

Immunohistochemical analysis for IP_3_R3 localization and classification of leukocytes was performed on lacrimal gland sections from wild-type, *Itpr3^−/−^*, and *Itpr2^−/−^;Itpr3^−/−^* mice. The extracted lacrimal glands were embedded in an optimal cutting temperature compound. The frozen sections (5-µm thick) were fixed with 10% formalin neutral buffer solution (Wako) and incubated with antibodies against IP_3_R3 (1∶250; BD Transduction Laboratories, Heidelberg, Germany), CD45, F4/80, CD19, CD8, or CD4 (1∶100; eBioscience, San Diego, CA, USA). Signals were detected by incubating with rabbit anti-mouse IgG antibodies conjugated with Alexa 488 or peroxidase (Dako, Glostrup, Denmark). Peroxidase-conjugated antibodies were visualized by adding diaminobenzidine tetrahydroxychloride. Nuclear staining was performed with 4′,6-diamidino-2-phenylindole (DAPI; Dojindo, Kumamoto, Japan) or hematoxylin.

### Measurement of Acinar Cell Area of the Lacrimal Glands

For quantitative analysis, hematoxylin/eosin (HE)-stained sections of the lacrimal glands from wild-type and *Itpr2^−/−^;Itpr3^−/−^* mice were used. The lacrimal acinar cell area was measured as reported previously [10].

### Measurement of Intracellular Ca^2+^ Concentration in Lacrimal Gland Cell Suspensions

Following deep anesthesia by the intraperitoneal injection of 60 mg/kg nembutal (Dainippon Sumitomo Pharma, Osaka, Japan), the mice were sacrificed. Subsequently, the exorbital lacrimal glands were immediately removed, placed in cold balanced salt solution (BSS) containing 115 mM NaCl, 5.4 mM KCl, 2 mM Ca^2+^, 1 mM Mg^2+^, 20 mM Hepes, and 10 mM glucose (pH7.4), and rapidly minced under exposure to 2 mg/mL collagenase type 2 (Worthington, Malvern, PA, USA) in BSA. The material was then digested for 10 min at 37°C with 2 mg/mL of collagenase type 2 in BSS, the suspension being gently passed through a pipette several times. After the digestion, 1 mL of BSS was added to the preparation and then centrifuged at 100×*g* for 3 min. The pellet was rinsed in 1 mL BSS and centrifuged in order to collect the lacrimal gland cells.

The isolated lacrimal gland cell preparation was incubated in 5 µM fura-2 AM (Dojindo)/BSS for 45 min at room temperature, rinsed twice, resuspended in 500 µL of BSS, and stored at 4°C. For the two-dimensional measurement of Ca^2+^ changes, a 75-µL sample of fura-2-loaded lacrimal gland cells was dispersed on a Cell-Tak (BD Biosciences, Bedford, MA, USA)-coated glass coverslip that formed the bottom of the recording chamber, mounted on the stage of an inverted fluorescein microscope (IX70, Olympus, Tokyo, Japan), and perfused with BSS at a rate of 2 mL/min at room temperature. Excitation of fura-2 was performed every 5 s by alternate illumination with 340 and 380 nm light. The resultant fluorescence (510–550 nm; F340/F380) was imaged using an objective lens (UPlanApo 20x/340, Olympus) and a silicon-intensified target camera to obtain pseudo-colored images of F340/F380, and stored in a personal computer using the ARGUS50/CA software (Hamamatsu Photonics, Shizuoka, Japan). The peak amplitude Ca^2+^ responses (R, delta Fura-2 ratio 340/380) were expressed as the averaged amplitude from 0–50 sec was equal to zero.

### Real Time RT-PCR

Total RNA was extracted from cells in the lacrimal glands of the mice using the TRIzol reagent (Invitrogen, Carlsbad, CA, USA) according to the manufacturer’s instructions. Complementary DNA was produced from total RNA using Superscript VILO™ Master Mix (Invitrogen). Quantitative real-time PCR was performed using the StepOne-Plus Real Time PCR system (Applied Biosystems) with Fast Advanced Master Mix (Applied Biosystems) and the predesigned primers for tumor necrosis factor alpha (TNF-α), interleukin-6 (IL-6), and glyceraldehyde-3-phosphate dehydrogenase (GAPDH) [TaqMan Gene Expression Assay (TNF-α: Mm00443258-m1, IL-6: Mm00446190-m1, and GAPDH: Mm99999915-g1)]. The mRNA levels were evaluated by the ΔΔCT method, and normalized to GAPDH mRNA.

### Enzyme-linked Immunosorbent Assay (ELISA) for Immunoglobulins and Auto-antibodies

The amounts of mouse immunoglobulins and auto-antibodies in sera from wild-type and *Itpr2^−/−^;Itpr3^−/−^* mice were analyzed by ELISA. For the detection of antibodies to SS-A antigens, the mouse sera were diluted 1∶100 and analyzed using mouse anti-SS-A IgG ELISA kits (Alpha Diagnostics, San Antonio, TX, USA).

### Statistical Analysis

All summarized data were expressed as means ± SEM. Statistical significance was calculated by unpaired Student’s *t*-test or Mann–Whitney *U*-test. A p value less than 5% was considered statistically significant.

## Results

### *Itpr2^−/−^;Itpr3^−/−^* Mice had Severe Defects in Tear Secretion Via Both Cholinergic and Adrenergic Receptor Pathways

**Figure 1:**
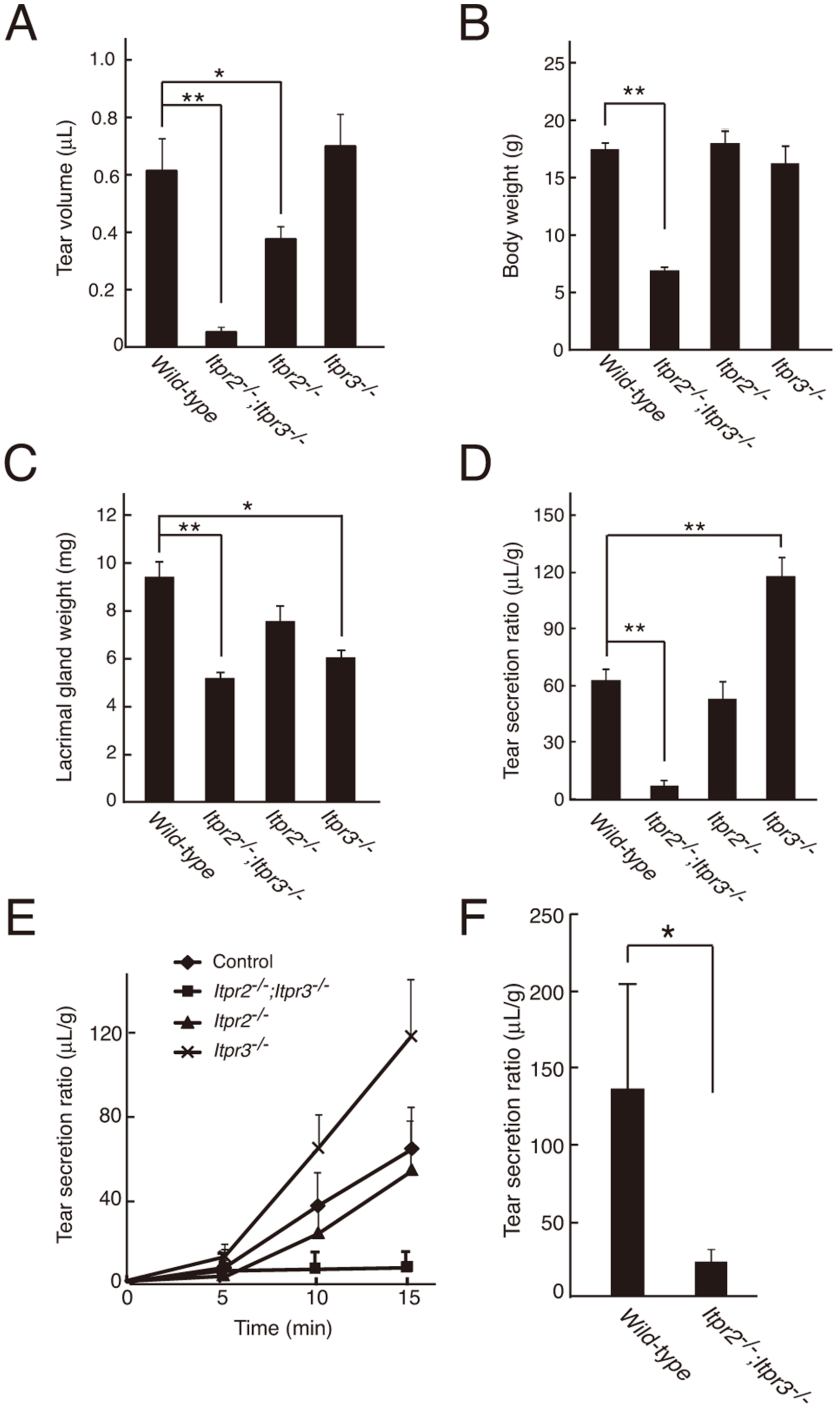
Defects in tear secretion in *Itpr2^−/−^;Itpr3^−/−^* mice via both parasympathetic and sympathetic pathways. (A) Tear volume in wild-type (n = 12) and *Itpr^−/−^* (n = 16) mice within 15 min of pilocarpine stimulation. (B) Average body weight of wild-type and the *Itpr^−/−^* mice at 6 weeks. (C) Average lacrimal gland weights of wild-type and the *Itpr^−/−^* mice. (D) Tear secretion by pilocarpine adjusted for the weight of each lacrimal gland. (E) Time course of tear secretion in each 5-min period after pilocarpine administration in wild-type (diamond), *Itpr2^−/−^* (triangle), *Itpr3^−/−^* (cross), and *Itpr2^−/−^*;*Itpr3^−/−^* (square) mice. (F) The tear secretion by epinephrine adjusted for weight of the each lacrimal gland. All data are presented as means ± standard error of the mean (SEM). Student’s t-test, *P<0.05; **P<0.01. All experiments were performed at least three times, and representative data are shown.

We have previously reported that IP_3_R2 and IP_3_R3 play crucial roles in secretions from salivary, pancreatic, and nasal glands [6], [7]. However, the subtypes of IP_3_R expressed in lacrimal glands and their contribution to tear secretion remain unknown. To analyze the role of IP_3_Rs in lacrimal glands, we measured tear flow in mice deficient in IP_3_Rs (Fig. 1A). Since the body weight and lacrimal gland weight were different between wild-type and mutant mice (Figs. 1B, 1C), the tear volume was normalized against lacrimal gland weight. After the intraperitoneal administration of pilocarpine, a cholinergic receptor agonist, wild-type mice shed a large volume of tears in a time-dependent manner (Fig. 1D, E). Tear secretion in *Itpr2^−/−^* mice was comparable with that in wild-type mice, while *Itpr3^−/−^* mice shed more tears than the wild-type mice. In contrast, tear secretion was abolished in *Itpr2^−/−^;Itpr3^−/−^* mice (Fig. 1D).

We also examined the contributions of IP_3_Rs in tear secretion via the sympathetic pathway. As shown in Fig. 1F, tear flow by intraperitoneal administration of epinephrine was clearly observed in wild-type mice, but not in *Itpr2^−/−^;Itpr3^−/−^* mice. These results suggest that IP_3_R2 and IP_3_R3 are the predominant subtypes of IP_3_Rs in lacrimal glands and are essential for tear secretion via both the cholinergic and sympathetic pathways.

### Acetylcholine- and Epinephrine-induced Ca^2+^ Signals are Abolished in *Itpr2^−/−^;Itpr3^−/−^* Lacrimal Acinar Cells

**Figure 2:**
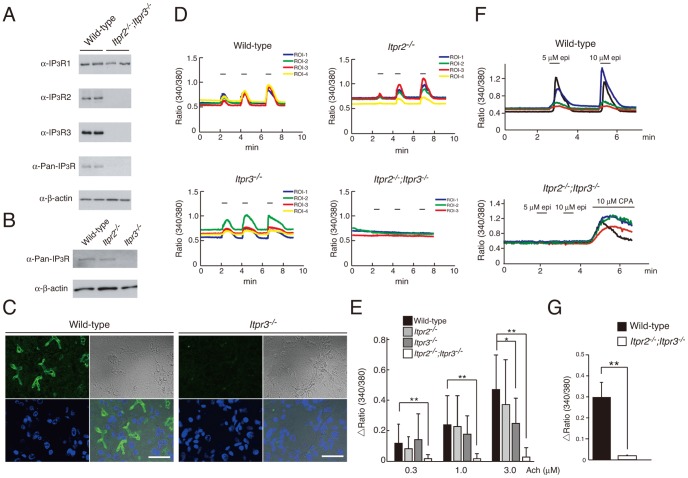
Lack of acetylcholine- and epinephrine-induced Ca^2+^ signals in lacrimal glands in *Itpr2^−/−^;Itpr3^−/−^* mice. (A and B) Western blot analysis of lacrimal glands from wild-type, *Itpr2^−/−^*, *Itpr3^−/−^*, and *Itpr2^−/−^*;*Itpr3^−/−^* mice, using IP_3_R antibodies. (C) Immunohistochemistry of IP_3_R3 in wild-type and *Itpr3^−/−^* lacrimal glands. Each panel indicates IP_3_R3 (green), DAPI (blue), visible image, and the merged image, respectively. Scale bar, 30 µm. All experiments were performed at least three times, and representative data are shown. (D) Dose-dependent Ca^2+^ response of lacrimal gland acinar cells. (E) Quantitation of Ca^2+^ peak amplitude. Lacrimal gland acinar cells were sequentially stimulated with 0.3, 1.0, and 3.0 µM acetylcholine. All data are presented as means ± SEM. Student’s t-test, *P<0.05; **P<0.01. All experiments were performed at least three times, and representative data are shown. (F) Ca^2+^ signals in response to the epinephrine (5, 10 µM) stimulation. Ten µM CPA, a SERCA pump inhibitor, was applied to check the Ca^2+^ store within the ER of *Itpr2^−/−^*;*Itpr3^−/−^* lacrimal acinar cells. (G) Quantitation of Ca^2+^ peak amplitude induced by 5 µM epinephrine.

We next examined the expression level of each IP_3_R subtype in the lacrimal glands. We found that all three types of IP_3_Rs were expressed in mouse lacrimal glands (Fig. 2A). No bands were detected with anti-Pan-IP_3_R antibodies in the *Itpr2^−/−^;Itpr3^−/−^* lacrimal gland lysates (Fig. 2A). In addition, IP_3_Rs were detected by anti-Pan-IP_3_R antibodies in lacrimal gland lysates from *Itpr2^−/−^* but not in *Itpr3^−/−^* mice (Fig. 2B), suggesting that IP_3_R3 exhibits the highest expression level among the three subtypes. Immunohistochemical studies using the anti-IP_3_R3 antibody revealed that IP_3_R3 is localized at the restricted region near the apical membranes in the acinar cells where endocrine secretion occurs (Fig. 2C). IP_3_R3 fluorescein staining was not detectable in *Itpr3^−/−^* mice (Fig. 2C).

Ca^2+^ transients were clearly observed in response to acetylcholine (Ach) in wild-type lacrimal gland acinar cells in a dose-dependent manner (Fig. 2D). The *Itpr2^−/−^* and *Itpr3^−/−^* acinar cells showed Ca^2+^ responses that were comparable to those of the wild-type cells, except that the *Itpr3^−/−^* cells exhibited relatively rather long-lasting Ca^2+^ signals with decreased peak amplitudes, especially at 3.0 µM Ach (Figs. 2D, 2E). These long-lasting Ca^2+^ signals were likely due to the nature of the residual IP_3_R2, which has the highest affinity for IP_3_ among the three types of IP_3_Rs, and might explain the larger amount of tear secretion in *Itpr3^−/−^* mice (Fig. 1D). In contrast, Ach-induced Ca^2+^ transients were diminished in the *Itpr2^−/−^;Itpr3^−/−^* acinar cells (Figs. 2D, 2E).

Moreover, *Itpr2^−/−^;Itpr3^−/−^* acinar cells exhibited no epinephrine-induced Ca^2+^ transients (Fig. 2F, G). The diminished Ca^2+^ signals in the *Itpr2^−/−^;Itpr3^−/−^* acinar cells on epinephrine stimulation was not due to the depletion of Ca^2+^ stores, because cyclopiazonic acid (CPA), a Ca^2+^ pump inhibitor, induced a considerable Ca^2+^ leak from the endoplasmic reticulum (Fig. 2F). These results suggest that IP_3_R2 and IP_3_R3 are essential for Ca^2+^ signals in both the sympathetic and parasympathetic pathways.

### *Itpr2^−/−^;Itpr3^−/−^* Mice cause Dry Eye

**Figure 3:**
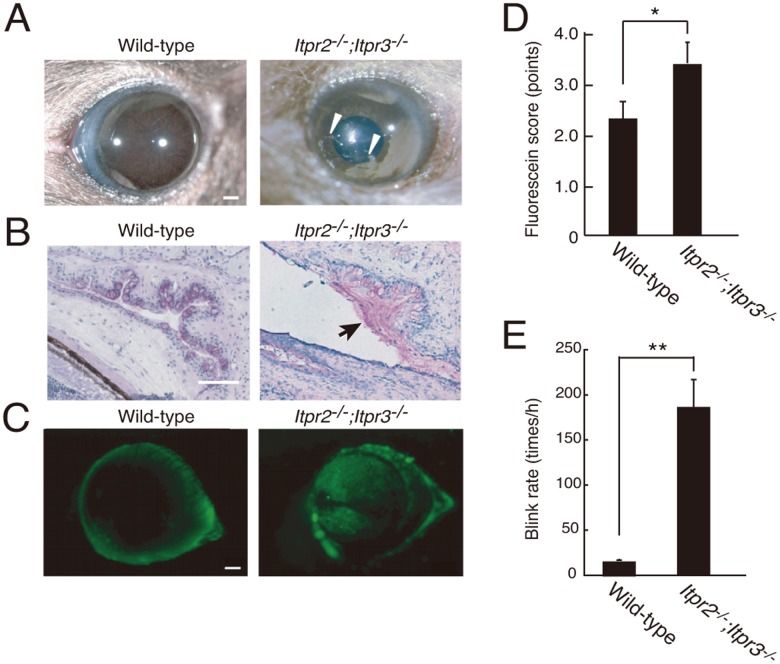
Altered ocular surface in *Itpr2^−/−^*;*Itpr3^−/−^* mice. (A) Anterior segment photos of the ocular surface. Wild-type and *Itpr2^−/−^*;*Itpr3^−/−^* mice corneas were viewed and photographed under white light. Debris is indicated by white arrowheads. Bar: 1 mm. (B) Histological detection of conjunctiva mucins stained with periodic acid-Schiff base. The conjunctiva of *Itpr2^−/−^*; *Itpr3^−/−^* mice had abundant mucin complexes (arrow head). Scale bar: 50 µm. (C, D) Anterior segment photos of ocular surface fluorescein staining, and the score. Bar: 1 mm. (E) Comparison of spontaneous blink rate. All data are presented as means ± SEM. Student’s t-test, *P<0.05. All experiments were performed at least three times, and representative data are shown.

We carefully checked the ocular surfaces of *Itpr2^−/−^;Itpr3^−/−^* mice. A significant amount of debris was observed on the corneal surfaces in *Itpr2^−/−^;Itpr3^−/−^* mice (Fig. 3A). Abnormalities of the conjunctival surface bound to abundant mucin complex were observed in *Itpr2^−/−^;Itpr3^−/−^* mice (Fig. 3B). A reduction in the number of goblet cells, a common feature of dry eye patients, was also observed in *Itpr2^−/−^;Itpr3^−/−^* mice. In addition, *Itpr2^−/−^;Itpr3^−/−^* mice showed increased corneal fluorescein staining at 6 weeks (Figs. 3C, D), which indicates corneal epithelial barrier disruption in these mutant mice. This was not due to the abnormal development of the corneal surface, because no significant difference was observed in corneal staining between the ocular surfaces of wild-type and *Itpr2^−/−^;Itpr3^−/−^* mice at 3 weeks after birth, immediately after the mice opened their eyes (data not shown). Moreover, *Itpr2^−/−^;Itpr3^−/−^* mice showed increased blink rates because of insufficient tear flow on the ocular surface (Fig. 3E).

### Atrophy of the Lacrimal Glands in *Itpr2^−/−^;Itpr3^−/−^* Mice

**Figure 4:**
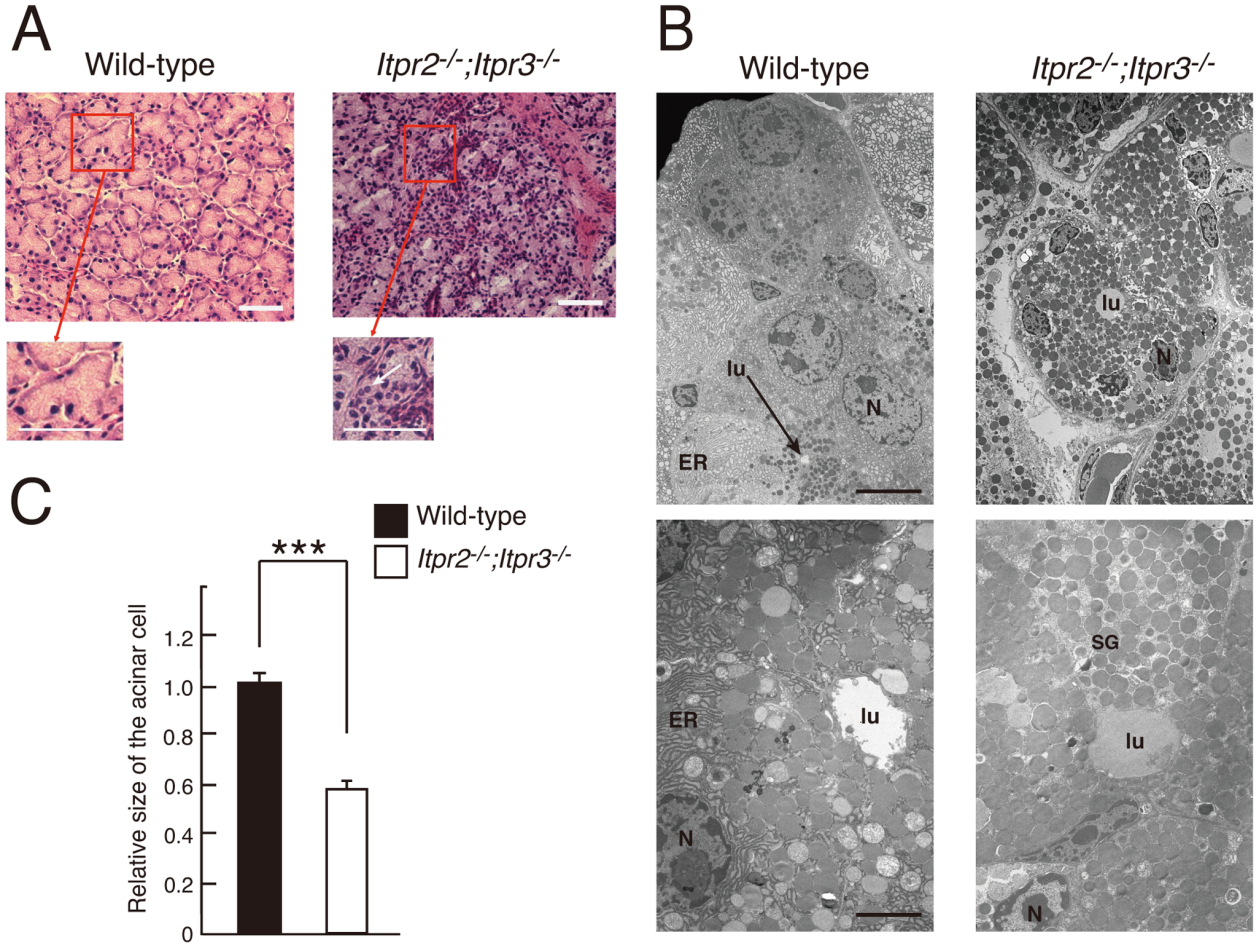
Histological analysis of lacrimal gland tissues. (A) Tissue sections of lacrimal glands from wild-type and *Itpr2^−/−^*;*Itpr3^−/−^* mice were stained by hematoxylin/eosin (HE) and observed under light microscopy. White arrowheads indicate inflammatory infiltrates. Scale bar: 50 µm. (B) Electron micrographs of lacrimal glands from wild-type and *Itpr2^−/−^*; *Itpr3^−/−^* mice. Scale bar: upper panels, 5 µm; lower panels, 2 µm. All experiments were performed at least three times, and representative data are shown. N; Nucleus, lu; lumen, ER; endoplasmic reticulum. (C) Relative lacrimal acinar cell area. The acinar cell area of wild-type (n = 54) and *Itpr2^−/−^*;*Itpr3^−/−^* (n = 59) lacrimal acinar cells was measured using HE-stained sections. Values represent the means ± SEM. Student’s t-test, ***, P<0.001.

We next performed histological analysis of the lacrimal gland tissues, and found atrophy of the lacrimal gland acinar units with marked lymphocytic infiltration in *Itpr2^−/−^;Itpr3^−/−^* mice more than 10 weeks of age (Fig. 4A). Electron micrographs also demonstrated the distinct morphology of acinar cells between wild-type and *Itpr2^−/−^;Itpr3^−/−^* mice. Secretory vesicles were located near the acinar lumen side and the well-developed endoplasmic reticulum (ER) structure was clearly observed in the cytoplasm near the apical side of the wild-type lacrimal acinar cells (Fig. 4B). In the *Itpr2^−/−^;Itpr3^−/−^* acinar cells, however, an excessive number of secretory vesicles accumulated and distributed in the cytoplasm, making it difficult to detect the ER in the cytoplasm (Fig. 4B). We also found that the *Itpr2^−/−^;Itpr3^−/−^* acinar cells seemed to be smaller than wild-type acinar cells. The lacrimal acinar cell area in *Itpr2^−/−^;Itpr3^−/−^* mice was approximately 40% smaller than that in wild-type mice (Fig. 4C).

### Inflammation of the Lacrimal Glands in *Itpr2^−/−^;Itpr3^−/−^* Mice

**Figure 5:**
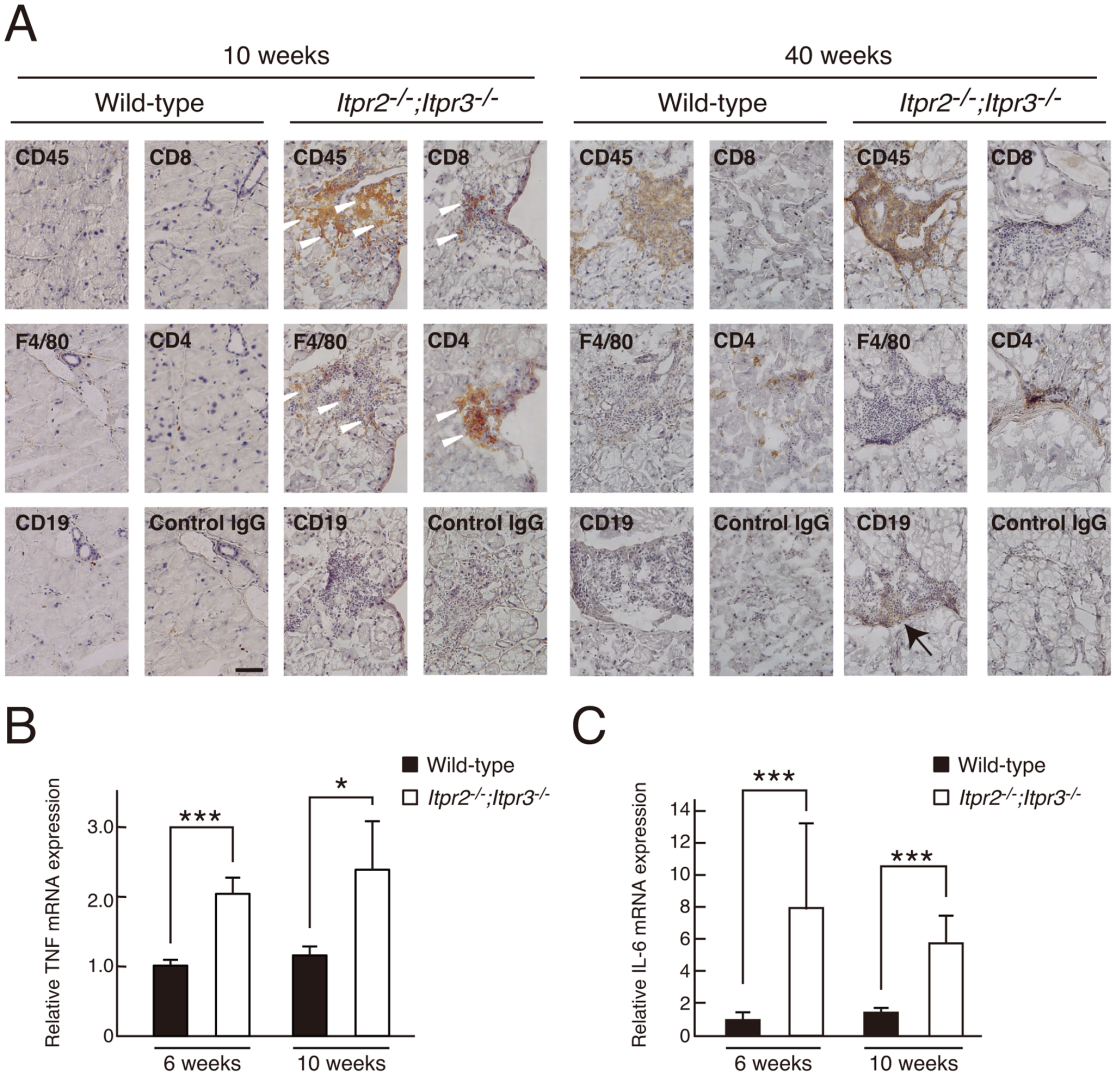
Infiltration of inflammatory mononuclear cells in *Itpr2^−/−^*;*Itpr3^−/−^* lacrimal glands. (A) Immunostaining of CD45, F4/80, CD19, CD8 and CD4 in lacrimal gland tissue sections from wild-type and *Itpr2^−/−^*;*Itpr3^−/−^* mice. White arrowheads indicate inflammatory mononuclear cells. (B) Quantification of TNF-α mRNA expression levels by real time RT-PCR. Six week-old mice; wild-type: n = 8 and *Itpr2^−/−^*;*Itpr3^−/−^*: n = 8. Ten week-old mice; wild-type: n = 16, *Itpr2^−/−^*;*Itpr3^−/−^*: n = 10. Mann–Whitney *U*-test, ***P<0.001, *P<0.05. All data are presented as means ± SEM. (C) Quantification of IL-6 mRNA expression levels by real time RT-PCR. Six week-old mice; wild-type: n = 8 and *Itpr2^−/−^*;*Itpr3^−/−^*: n = 8. Ten week-old mice; wild-type: n = 16, *Itpr2^−/−^*;*Itpr3^−/−^*: n = 10. Mann–Whitney *U*-test, ***P<0.001. All data are presented as means ± SEM.

To further explore the infiltration state of the lacrimal glands in *Itpr2^−/−^;Itpr3^−/−^* mice, we classified the inflammatory infiltrates by using several lymphocyte markers (leukocyte; CD45, macrophage; F4/80, T-cell; CD4 and CD8, B-cell; CD19). We found that CD45-positive inflammatory mononuclear cells infiltrated the lacrimal glands in *Itpr2^−/−^;Itpr3^−/−^* mice at 10 weeks (Fig. 5A, left panel, white arrow heads). These CD45-positive cells were located in the interstitial space around the lacrimal gland acinar cells. Macrophages and activated T-cells were the major inflammatory cells at 10 weeks (Fig. 5A); however, the population of infiltrating cells changed thereafter, and many B cells were detected at 40 weeks (Fig. 5A, right panel, arrow). We also checked the inflammatory environment of the lacrimal glands by evaluating the levels of pro-inflammatory cytokines. We found that the expression levels of pro-inflammatory cytokines such as TNF-α and IL-6 were significantly increased in the lacrimal glands in *Itpr2^−/−^;Itpr3^−/−^* mice (Fig. 5B and C).

### *Itpr2^−/−^;Itpr3^−/−^* Mice Present Autoantibodies against Ribonucleoprotein SSA

**Figure 6:**
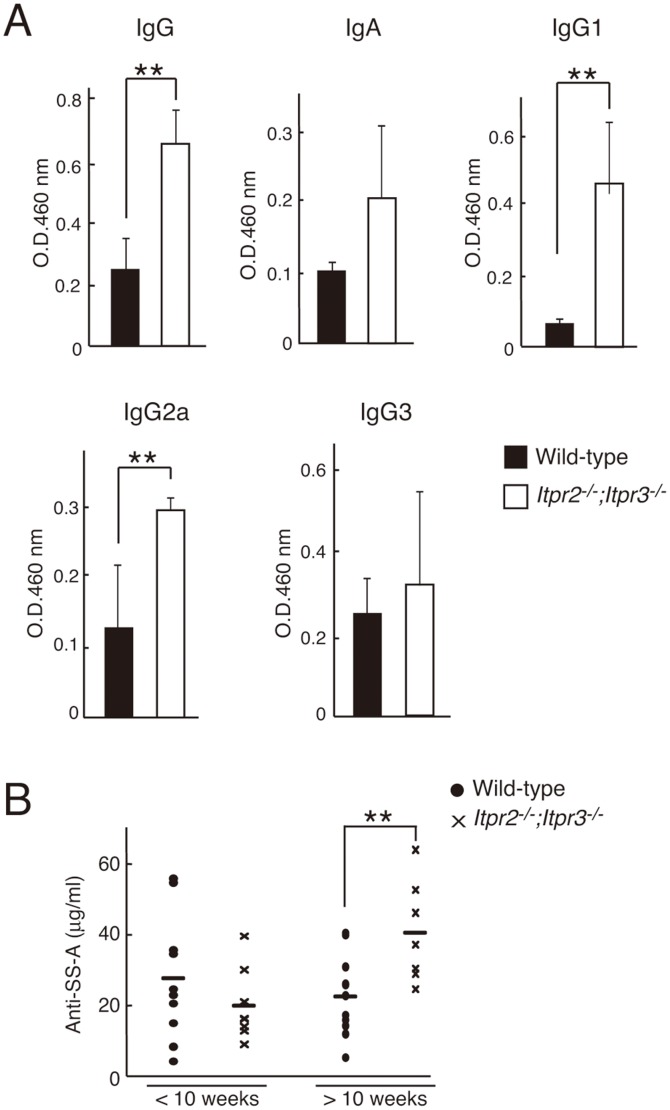
Elevation of serum immunoglobulins and autoantibodies to SS-A antigens in *Itpr2^−/−^*;*Itpr3^−/−^* mice. (A) Serum levels of immunoglobulins. Serum samples were collected from 8-week-old wild-type and *Itpr2^−/−^*;*Itpr3^−/−^* mice. Serum levels of IgG, IgA, IgG1, IgG2a, and IgG3 were measured by ELISA. (B) Serum levels of autoantibodies in wild-type (6 weeks, n = 10; 10–35 weeks, n = 11) and *Itpr2^−/−^*;*Itpr3^−/−^* (6 weeks, n = 8; 10–35 weeks, n = 7) mice. Serum levels of autoantibodies to SS-A antigens. Bars show the means. All data are presented as means ± SEM. Student’s *t*-test, *P<0.05. All experiments were performed at least three times, and representative data are shown.

We finally examined the concentrations of immunoglobulins and autoantibodies against ribonucleoprotein SSA, one of the most commonly detected autoantibodies in patients with SS, in the serum of *Itpr2^−/−^;Itpr3^−/−^* mice. As shown in Fig. 6A, we found that the concentration of immunoglobulin was significantly higher in *Itpr2^−/−^;Itpr3^−/−^* mice than in wild-type mice. Moreover, the levels of autoantibodies against SSA were significantly higher in *Itpr2^−/−^;Itpr3^−/−^* mice compared to wild-type mice at 10 weeks, when the infiltrates were observed (Fig. 6B).

## Discussion

In this study, we have shown that the type 2 and type 3 IP_3_Rs are predominantly expressed in lacrimal glands and that IP_3_Rs are essential for tear secretion via both the sympathetic and parasympathetic signaling pathways. We also found that Ca^2+^ signals in response to epinephric as well as cholinergic receptors were diminished in *Itpr2^−/−^;Itpr3^−/−^* lacrimal gland cells. The lack of tear flow resulted in increased eye blink rates, and the corneal surface and conjunctiva were severely damaged in *Itpr2^−/−^;Itpr3^−/−^* mice. As the mutant mice aged, *Itpr2^−/−^;Itpr3^−/−^* mice displayed atrophy and infiltration of lacrimal glands as well as the production of autoantibodies against SSA in the sera, which are clinical features observed in human SS [11], [12]. Thus, our *Itpr2^−/−^;Itpr3^−/−^* mice constitute a novel dry eye mouse model with an SS-like phenotype.

It is well known that norepinephrine released from sympathetic nerves predominantly activates α1-adrenergic receptors and induces Ca^2+^ elevation in lacrimal acinar cells [13]. However, in contrast to the established role of IP_3_R in Ca^2+^ elevation induced by parasympathetic stimuli, the Ca^2+^ channels that are responsible for cytosolic Ca^2+^ elevation triggered by α-adrenergic stimuli are not clearly identified in lacrimal acinar cells. Several previous studies suggested a role for ryanodine receptors in Ca^2+^ elevation in lacrimal glands by norpinephrine [3]. Our study clearly demonstrated that IP_3_Rs contribute significantly to adrenergic tear secretion as well as cholinergic tear secretion *in vivo*. Ca^2+^ transients triggered by epinephrine were diminished in *Itpr2^−/−^;Itpr3^−/−^* lacrimal gland acinar cells. These results suggest that Ca^2+^ release from IP_3_Rs is a crucial event in both cholinergic and adrenergic signal transduction in lacrimal glands, which underlies the lack of tear secretion, resulting in the abnormal ocular surface seen in *Itpr2^−/−^;Itpr3^−/−^* mice.

It is an important observation that *Itpr2^−/−^;Itpr3^−/−^* mice developed only corneal and conjunctival injuries at 6 weeks of age and showed lacrimal gland infiltrations only after 10 weeks of age. Thus, ocular surface disturbance seems to occur prior to lymphocyte infiltration into the lacrimal glands in *Itpr2^−/−^;Itpr3^−/−^* mice. Together with the previous finding that the desiccating stress of the ocular surface induces lacrimal gland inflammation and infiltration [14], corneal surface and conjunctival injuries caused by long-lasting dysfunction of lacrimal acinar cells may lead to the activation of antigen-presenting cells [15] and the subsequent breakdown of self-tolerance against endogenous epitopes shared among lacrimal gland units. Further studies are necessary for a clear understanding of the mechanism of infiltration in the lacrimal glands, which might contribute to the pathogenesis of SS in humans.

In conclusion, we have demonstrated that IP_3_R2 and IP_3_R3 play a central role in tear secretion and maintenance of the lacrimal glands. Our data indicate that Ca^2+^ release from IP_3_Rs in lacrimal gland acinar cells is essential for sympathetic as well as cholinergic tear secretion. Together with the defect in saliva secretion observed in our previous study [6], the diversified symptoms of *Itpr2^−/−^;Itpr3^−/−^* mice including lacrimal gland inflammatory foci, ocular surface disruption, and the production of autoantibodies against SSA fulfill the criteria for a diagnosis of SS, established by the American-European Consensus Group [16]. We believe that *Itpr2^−/−^;Itpr3^−/−^* mice will be a useful tool for the analysis of pathological mechanisms and for the development of new treatment strategies for SS.
